# Supplementary material for: Sex differences in microglial CX3CR1 signalling determine obesity susceptibility in mice
Source: Nat Commun. 2017 Feb 22;8:14556. doi: 10.1038/ncomms14556 (PMC5322503; doi:10.1038/ncomms14556)
Supplement: Supplementary Information — Supplementary Figures. [file ncomms14556-s1.pdf]

Supplementary Information

Supplementary Figure 1

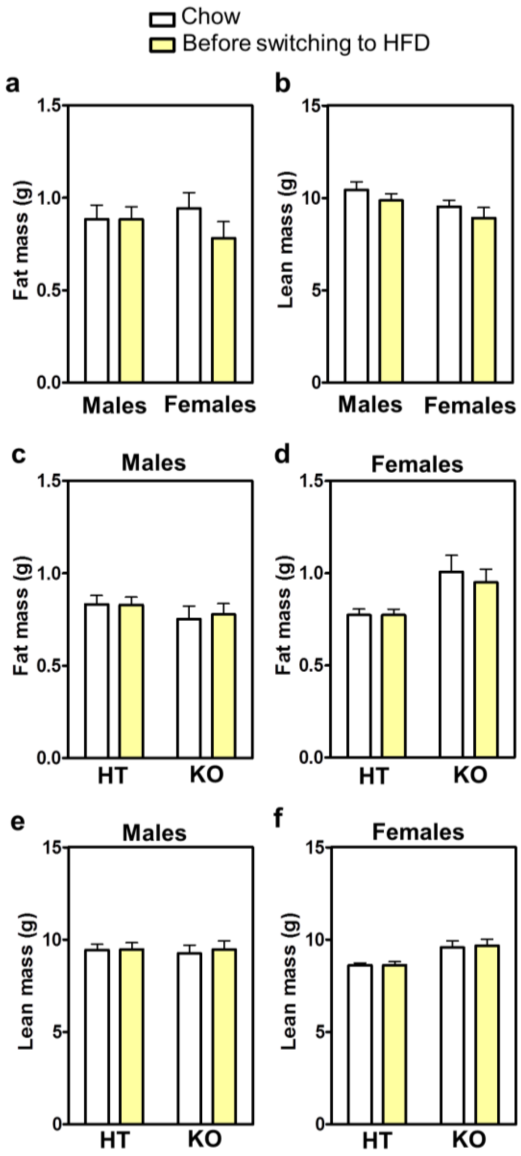

Supplementary Figure 1. Initial body composition of mouse cohorts.
(**a,b**) Total fat mass (**a**) and lean mass (**b**) in wild-type male and female mice depicted in Fig. 1a and 1b, 2 weeks prior to diet switch. White bars represent mice remaining on chow while yellow bars indicate mice subsequently placed on HFD. n = 12 males and 8 females per group. (**c-f**) Total fat mass (**c,d**) and lean mass (**e,f**) in Cx3cr1 HT and KO males (**c,e**), and females (**d,f**) depicted in Fig. 2a and 2b, 2 weeks prior to diet switch. White bars represent mice remaining on chow while yellow bars indicate mice subsequently placed on HFD. n = 12 males and 8 females per group. Bars represent mean ± SEM.

Supplementary Figure 2

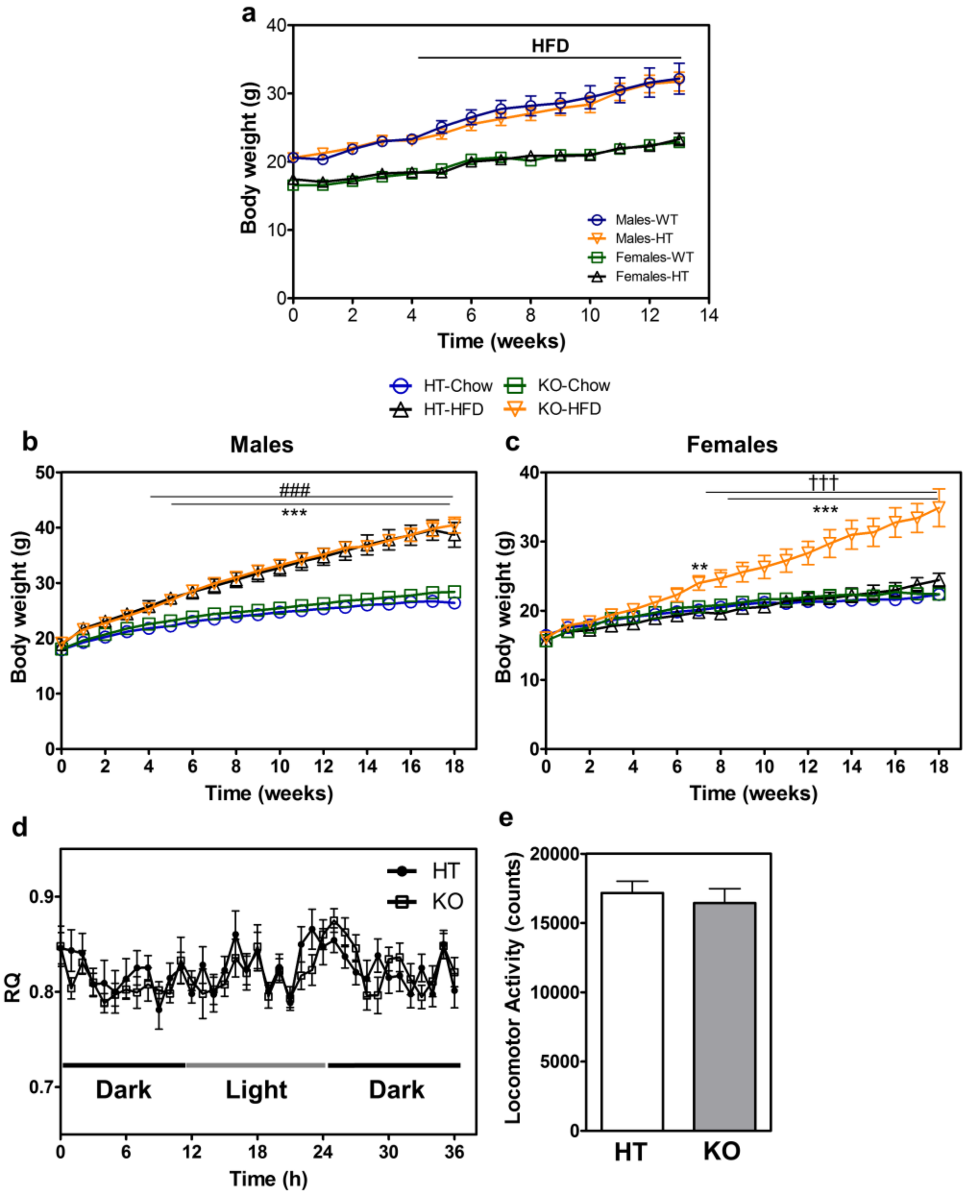

Supplementary Figure 2. HFD-fed female Cx3cr1 KO mice exhibit an obesity phenotype with no alterations of respiratory quotient or ambulatory activity.

(a) Groups of male and female HT and wild-type (WT) mice were weighed weekly for 4 weeks on normal chow followed by switch to HFD for an additional 9 weeks. Each data point represents mean ± SEM of 5 animals per group. **(b,c)** Absolute body weight measured in Cx3cr1-heterozygous (HT) and knockout (KO) mice on chow and HFD for 18 weeks (panel b: males; panel c: females). Data are presented as mean ± SEM of 12 males and 8 females per group *p<0.05, **p<0.01 and ***p<0.001 vs chow groups; ###p<0.001 HT-HFD compared to HT chow. †††p<0.001 KO-HFD vs HT-HFD. (**d**) Respiratory quotient (RQ) measured continuously over 36 hrs (2 dark cycles and 1 light cycle) in HT and KO female mice exposed to HFD for 18 weeks. Each point represents mean ± SEM of 8 animals per group. (**e**) Daily average locomotor activity measured as number of beam breaks in HT and KO female mice exposed to HFD for 18 weeks. Bars are mean ± SEM of 8 animals per group.

Supplementary Figure 3

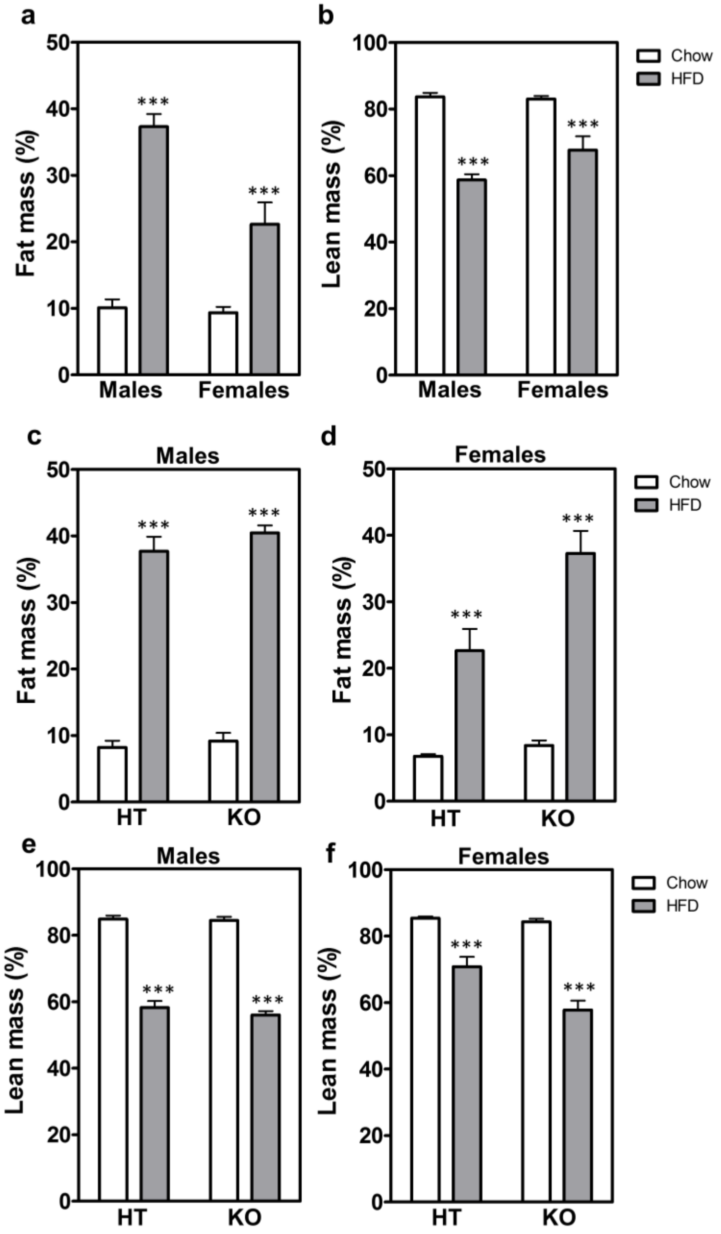

Supplementary Figure 3. HFD feeding increases percent fat mass in males and *Cx3cr1*-deficient females. (**a,b**) Percent fat mass (**a**) and lean mass (**b**) in chow-fed and HFD groups. **(c-f)** Percent fat mass (**c,e**) and lean mass (**e,f**) measured at study end in HT and KO mice (panel c, e: males; panel d, f: females). Data are presented as mean ± SEM of 12 males and 8 females per group. For all panels, data are analyzed by repeated measures or 2-way ANOVA followed by Bonferroni post-hoc comparisons. ***p<0.001 vs chow groups.

Supplementary Figure 4

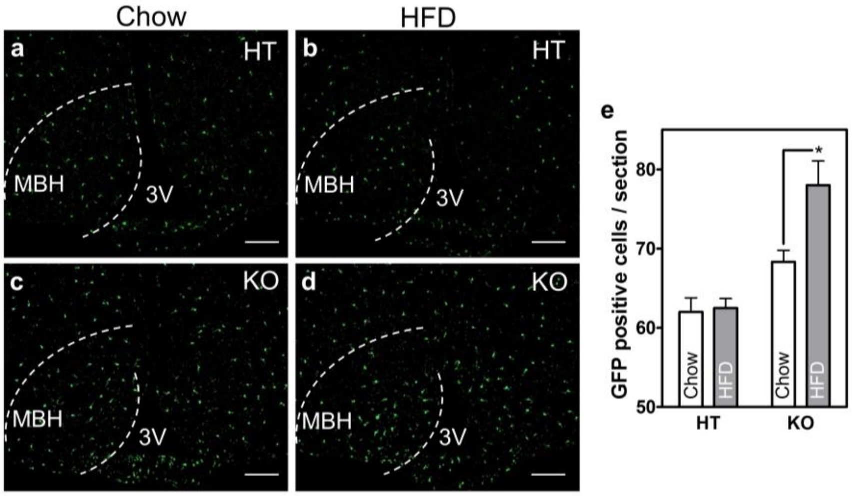

Supplementary Figure 4. *Cx3cr1*-deficient female mice display HFD-induced hypothalamic microglial accumulation. (**a-d)** Representative images showing CX3CR1-positive (GFP+) cells in the mediobasal hypothalamus (MBH) of female mice. 3V = third ventricle. (**e**) Quantification of GFP-positive cells in bilateral MBH from 6 sections per animal. Mean ± SEM, n = 4 per group. Scale bar 50 µm. *p<0.05

Supplementary Figure 5

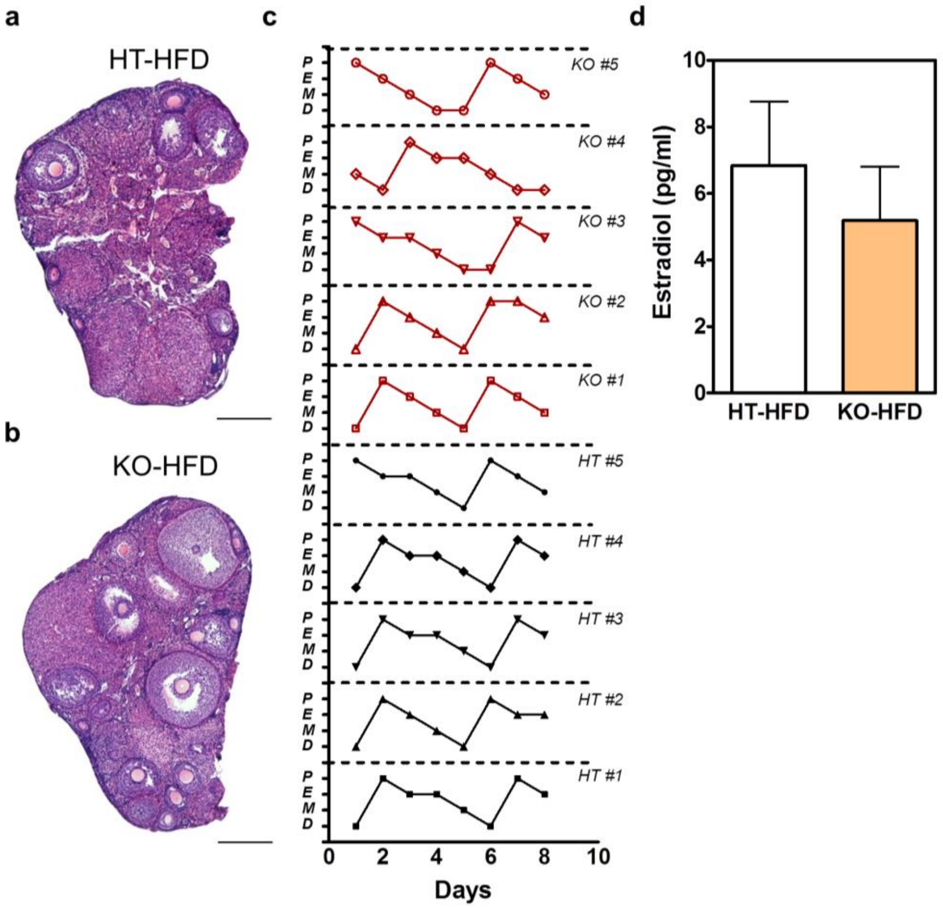

Supplementary Figure 5. Cx3cr1 KO female mice do not exhibit ovarian dysfunction.
(**a,b**) Representative H&E-stained sections from HT and KO mouse ovaries after 18 weeks of HFD feeding. n = 3 per group. Scale bar = 200 µm. (**c**) The estrous cyclicity of HT (black lines) and KO (red lines) was recorded over 8 days after 18 weeks of HFD exposure. P, proestrus; E, estrus; M, diestrus day 1; D, diestrus day 2. n = 5 per group. (**d**) Serum estradiol levels measured during diestrus in HT and KO mice exposed to HFD. Bars are mean ± SEM of 8 animals per group.

Supplementary Figure 6

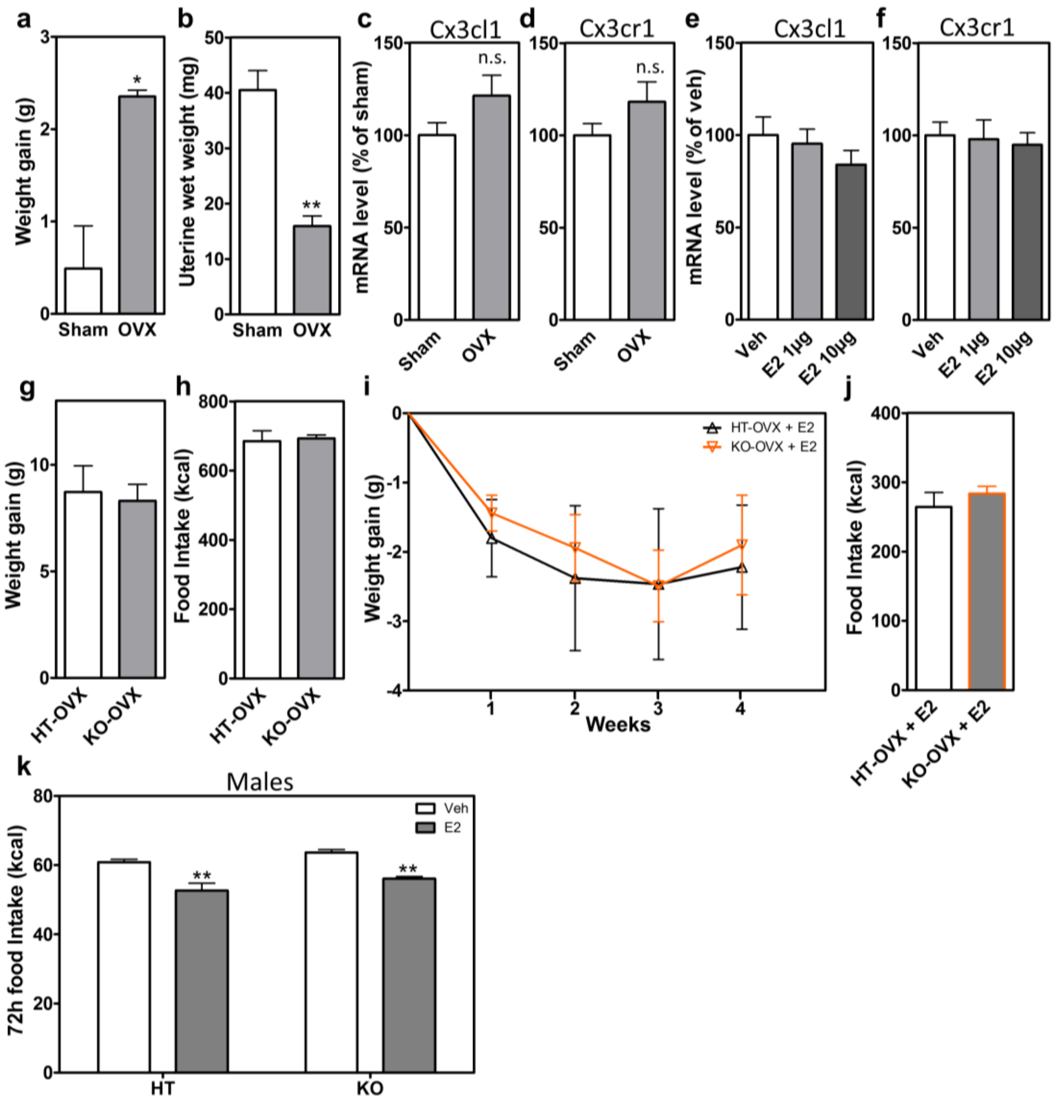

Supplementary Figure 6. Estrogen does not mediate sex-specific protection from DIO through the CX3CR1 signaling system.

(a) Body weight gain in sham-operated and OVX females fed with HFD for 2 weeks. **(b)** Uterine wet weight in sham and OVX mice. **(c-d)** Hypothalamic mRNA level of *Cx3cl1* (c) and *Cx3cr1* (d) of OVX and sham mice from a-b. **(e-f)** Hypothalamic mRNA level of *Cx3cl1* (e) and *Cx3cr1* (f) in HFD-fed male mice 2 hours after estradiol (E2) administration. **(g-h)** Total body weight gain (g) and cumulative food intake (h) in *Cx3cr1* HT and KO OVX mice exposed to HFD for 9 weeks. **(i-j)** Body weight gain (i) and cumulative food intake (j) during 4 weeks of continuous s.c. E2 administration in HT and KO OVX mice. **(k)** 72 hour HFD intake in HT and KO males receiving a single administration of E2 10mg s.c. Bars are mean ± SEM of 6 animals per group. *$p<0.05$ and **$p<0.01$.

Supplementary Figure 7

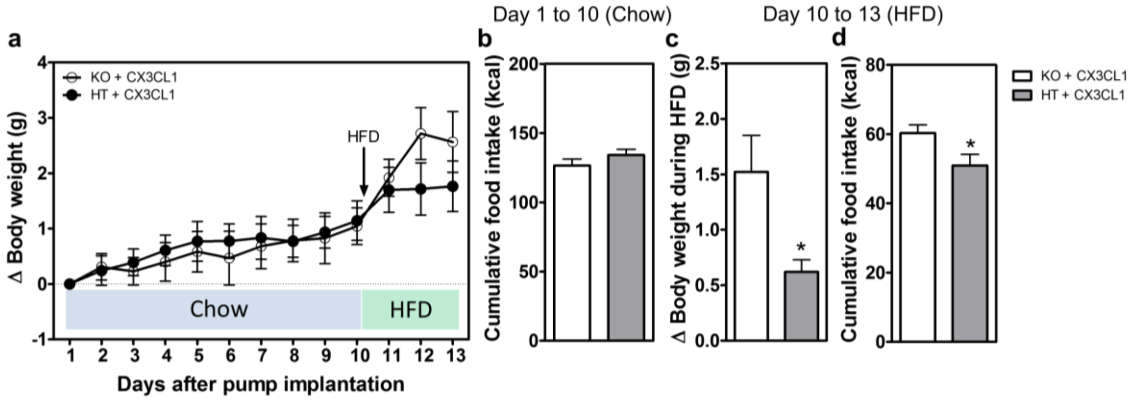

Supplementary Figure 7. Central administration of CX3CL1 does not alter body weight gain on a chow diet. (**a**) Weight gain in chow-fed *Cx3cr1* KO and HT male mice infused ICV with CX3CL1 (500ng/day) over 10 days and switched to HFD for the last 3 days of the infusion. (**b**) Cumulative food intake over the first 10 days of CX3CL1 ICV infusion (day 1 to 10; Chow). (**c,d**) Body weight gain (**c**) and cumulative food intake (d) over the last 3 days of CX3CL1 ICV infusion (day 10 to 13; HFD). Mean ± SEM, n = 6 animals per group. *$p<0.05$.
